# Efficacy of exhaustive and fixed-time drainage for chronic subdural hematoma after burr hole craniostomy

**DOI:** 10.12669/pjms.41.10.12663

**Published:** 2025-10

**Authors:** Jun Gu, Ya Xu, Qunfeng Gui, Huaqun Chen

**Affiliations:** 1 Jun Gu, Department of Neurosurgery, Yancheng Third People’s Hospital, The Yancheng School of Clinical Medicine of Nanjing Medical University, Yancheng, Jiangsu Province 224000, P.R. China; 2 Ya Xu, Department of Neurosurgery, Yancheng Third People’s Hospital, The Yancheng School of Clinical Medicine of Nanjing Medical University, Yancheng, Jiangsu Province 224000, P.R. China; 3 Qunfeng Gui, Department of Neurosurgery, Yancheng Third People’s Hospital, The Yancheng School of Clinical Medicine of Nanjing Medical University, Yancheng, Jiangsu Province 224000, P.R. China; 4 Huaqun Chen, Department of Neurosurgery, Yancheng Third People’s Hospital, The Yancheng School of Clinical Medicine of Nanjing Medical University, Yancheng, Jiangsu Province 224000, P.R. China

**Keywords:** Burr hole craniostomy, Chronic subdural hematoma, Drainage, Exhaustive, Fixed time

## Abstract

**Objective:**

To compare the efficacy of exhaustive and fixed-time drainage for chronic subdural hematoma (CSHD) after burr hole craniostomy (BCT).

**Methodology:**

This single-center retrospective case-control study included 140 patients with CSHD who underwent BCT at the Third People’s Hospital of Yancheng City from December 2021 to April 2024. According to the surgical records, 69 patients underwent fixed-time drainage, which was removed after 48 hours (control group) and 71 patients underwent postoperative exhaustive drainage (observation group). The primary outcomes of interest included surgical efficacy and recurrence rate at six months after surgery. The secondary outcomes were Markwalder’s Grading Scale and Glasgow Coma Scale (MGS-GCS), modified Rankin scale (mRS) score and incidence of complications.

**Results:**

Six months after surgery, the total efficacy of the observation group (91.5%) was significantly higher than that of the control group (79.7%), while the recurrence rate was significantly lower (4.2% vs. 14.5% in the control group) (P<0.05). The MGS-GCS grading of both groups improved compared to preoperative levels and was significantly better in the observation group than the control group (P<0.05). Intervention led to a decrease in the mRS scores of both groups. The post-intervention mRS scores in the observation group were considerably lower than in the control group (P<0.05). The incidence of complications in the observation group (9.9%) was lower than that in the control group (20.3%), but the difference was not statistically significant (P>0.05).

**Conclusions:**

Compared with fixed-time drainage, exhaustive drainage of CSDH after BCT can improve surgical efficacy and reduce the recurrence rate. Further multi-center randomized controlled trials are still needed to confirm the results.

## INTRODUCTION

Chronic subdural hematoma (CSDH), the accumulation of blood and fluid between the arachnoid membrane and dura mater, often leads to the compression of the adjacent brain tissue and varying degrees of neurological dysfunction, including cerebral herniation.^1^-^3^ The incidence of CSDH is 1.72 to 20.6 per 100000 people per year and continues to rise with the aging of the population.^2^-^4^ Surgical intervention is the most commonly used treatment method for CSDH.^2^,^5^,^6^ Burr hole craniostomy (BCT) is the drilling drainage technique with postoperative conventional drainage, first proposed by Santarius et al. from the University of Cambridge in 2009. BCT relies on preoperative cranial computed tomography (CT) to guide drilling and drainage at the site of maximal hematoma thickness. During the operation, the hematoma cavity is thoroughly rinsed with physiological saline, followed by the placement of a drainage tube. After the surgery, natural drainage is performed for 48 hours before the removal of the tube.^6^

This treatment strategy has the advantages of minimal surgical trauma, standardized and straightforward operation and short hospitalization time.^6^,^7^ However, BCT has certain limitations, such as a lack of individualized measures during the treatment process. As the optimal postoperative drainage times after surgery are often unclear, some patients may experience insufficient hematoma drainage and residual hematoma.^8^ Reports are confirming that residual hematoma is an independent risk factor for postoperative recurrence in CSDH, leading to some patients requiring secondary surgery.^9^,^10^ Therefore, minimizing residual hematoma after BCT is an important measure to reduce the recurrence rate of CSDH.^8^-^10^

Combining BCT with postoperative exhaustive drainage with urokinase flushing is an effective method for promoting hematoma discharge.^11^ Urokinase is a thrombolytic agent that has been successfully used for liquifying hematomas.^12^ With this approach, the timing of drainage termination can be personalized based on the results of a follow-up head CT scan, thereby reducing the recurrence rate and improving the patient’s overall prognosis.^11^,^13^

This retrospective study compared the efficiency of exhaustive drainage with urokinase flushing and fixed postoperative drainage. The study aimed to provide reference suggestions for drainage strategies following BCT in CSDH.

## METHODOLOGY

This single-center retrospective case-control study included 140 CSDH patients who underwent BCT at the Third People’s Hospital of Yancheng City from December 2021 to April 2024. According to the surgical records, 69 cases underwent drainage removal after a fixed 48-hour period (control group); 71 cases underwent postoperative exhaustive drainage (observation group).

### Ethical approval:

The ethics committee of our hospital approved this study with the number YCSYLL2024163 at December 23, 2024.

### Inclusion criteria:

- Diagnosed with chronic subdural hematoma (CSDH) according to combined clinical and cranial CT criteria, consistent with the recommendations of the World Federation of Neurosurgical Societies (WFNS) and other recognized guidelines.^1^
- 18 years old ≤ 90 years old.
- Preoperative neurological dysfunction, such as memory loss, delayed response, aphasia, unclear consciousness, limb convulsions, unstable walking, limb numbness, vomiting and nausea, headache and dizziness, etc.
- Underwent drilling and drainage treatment.
- Complete clinical data.
- In this study, the diagnosis of CSDH required: (1) characteristic clinical manifestations such as headache, cognitive changes, limb weakness, or speech disturbance; (2) cranial CT findings showing hypodense, isodense, or mixed-density crescent-shaped collections in the subdural space; and (3) exclusion of acute or subacute subdural hematoma and other intracranial pathologies.

### Exclusion criteria:

- Previous history of intracranial surgery.
- Individuals with immune and hematological disorders.
- Recurrent/acute subdural hematoma.
- Individuals with other brain diseases that require surgical treatment.
- Patients who are expected to be unable to complete regular follow-up within 6 months due to various reasons.

### Drilling and drainage surgery:

All patients were guided to lie on their side and local anesthesia was administered. Preoperative cranial CT was referred to incise the scalp at the maximum level of hematoma. A bone hole (diameter 1.5 cm) was made to expose the dura mater. Bipolar electrocoagulation hemostasis and cross incision of the dura mater were done. A drainage tube was inserted into the hematoma cavity and the hematoma cavity was thoroughly irrigated with 1000 mL of room-temperature sodium chloride through the drainage tube until the effluent appeared clear. Subsequently, the head of the drainage tube was fixed to the patient’s forehead facing the hematoma cavity with a maximum diameter of about 1/2, the incision was closed layer by layer and the drainage bag was connected. The drainage bag was fixed to the bedside below the head for natural drainage. All operations were performed by experienced attending neurosurgeons from the same department, strictly adhering to a standardized burr-hole craniostomy protocol, including uniform patient positioning, burr-hole location, irrigation volume, drainage tube placement, and postoperative drainage management.

### Fixed time drainage:

During the operation, the hematoma cavity was thoroughly flushed with physiological saline and a drainage tube was placed inside the hematoma cavity. After natural drainage, the drainage tube was removed 48 hours after surgery.

### Exhaustive drainage:

A follow-up cranial CT scan was performed on the first day after surgery. If there was no high-density residual hematoma and the maximum transverse diameter was less than 3-mm, the drainage tube was removed after natural cessation, which, according to our institutional protocol, was defined as a 24-hour output of less than approximately 20–30 mL combined with follow-up cranial CT imaging showing no appreciable high-density residual hematoma requiring further evacuation. Otherwise, 20000 units of urokinase (Science Sun, China) were injected into the hematoma cavity through a drainage tube to promote drainage. A physiological saline (5 ml) was used to flush the tube and the drainage tube was closed for two hours and then reopened. After the drainage stopped, the cranial CT was repeated. In cases of high-density residual hematoma and the maximum transverse diameter of greater than 3 mm, the injection of urokinase and drainage were repeated. If the urokinase flush was performed three times, the drainage tube was removed once the drainage stopped, thereby terminating the treatment.

### Urokinase safety screening and aseptic administration:

Before instillation, all patients were evaluated for contraindications to urokinase, including active intracranial or systemic bleeding, recent major surgery, severe coagulopathy (e.g., INR > 1.5 or platelet count < 100 × 10^9^/L), uncontrolled hypertension, and any documented hypersensitivity to urokinase or other fibrinolytics. Allergy history was specifically reviewed for prior adverse reactions to fibrinolytic agents. Urokinase was reconstituted immediately before use and instilled through the drainage catheter under strict aseptic technique by experienced neurosurgical staff, using sterile gloves, drapes, and single-use syringes, with closed-system connection to minimize infection risk.

### Outcomes of interest:

The primary outcomes of interest included;

- Surgical effect: Marked effect: disappearance of adverse signs and clinical symptoms, complete clearance of subdural hematoma; Effective: Adverse signs and clinical symptoms have been alleviated and subdural hematoma has been completely cleared; Invalid: There is no change in adverse signs and clinical symptoms, but hematoma or hematoma enlargement still exists; Total effective rate= (significant effect + effective)/total number of cases × 100%.
- Recurrence rate. Recurrence was determined if, within six months after surgery, the patient’s head CT confirms the presence of hematoma, accompanied by relevant clinical symptoms and requires further surgical treatment.

### The secondary outcomes were:

- Consciousness state, evaluated using Markwalder’s Grading Scale and Glasgow Coma Scale (MGS-GCS), with levels ranging from 0 to 4. The higher level indicates a worse consciousness state and a more severe neurological impairment.
- Neurological function, scored according to the modified Rankin Scale (mRS) evaluation, with a score ranging from 0 to 6. A lower score indicates better neurological function.
- The occurrence of complications, including infection, subdural fluid accumulation, cerebrospinal fluid leakage, subdural gas accumulation, etc.

### Statistical analysis:

All statistical analyses were performed using STATA version 12.0 (StataCorp, College Station, TX, USA). The Kolmogorov–Smirnov test was used to assess the normality of continuous variables. Normally distributed data (e.g., age, hematoma volume) were expressed as mean ± standard deviation, while non-normally distributed data (e.g., mRS scores) were expressed as median and interquartile range (IQR). Continuous variables were compared between groups using the independent-samples t-test for normally distributed data or the Mann–Whitney U test for non-normally distributed data; paired comparisons within groups were assessed using the Wilcoxon signed-rank test. Categorical variables were expressed as counts and percentages n (%) and compared using the chi-square test or Fisher’s exact test when expected cell counts were <5. A two-tailed P-value <0.05 was considered statistically significant. Propensity score matching or multivariable logistic regression was not performed because the modest total sample size and the limited number of outcome events (recurrence and complications) did not meet the recommended events-per-variable thresholds for stable adjustment models; therefore, unadjusted between-group comparisons are reported.

## RESULTS

A total of 140 patients were included in this study. Of them, 85 were males, accounting for 60.7%. The age range of patients was 39-89 years, with an average of 66.4 ± 11.3 years. There was no significant difference in the baseline data between the two groups of patients (P>0.05) (Table-I). Six months after surgery, 43 patients in the observation group showed a marked improvement and the intervention was considered effective in 22 patients. The total effective rate (91.5%) of the observation group was significantly higher than that of the control group (79.7%) (P<0.05); The recurrence rate of the observation group was 4.2% (3/71), significantly lower than the control group’s 14.5% (10/69) (Table-II).

**Table-I:** Comparison of baseline data between two groups.

Baseline data	Observation group (n=71)	Control group (n=69)	t/χ^2^	P
Age, mean±SD	67.3±10.3	65.4±12.3	1.016	0.312
Male (yes), n(%)	42 (59.2)	43 (62.3)	0.147	0.702
Hematoma volume (ml), mean±SD	70.7±17.4	67.6±16.5	1.055	0.293
Hematoma location, n(%)			1.841	0.398
Left	35 (49.3)	28 (40.6)		
Right side	23 (32.4)	30 (43.5)		
Bilateral	13 (18.3)	11 (15.9)		
Diabetes (yes), n(%)	19 (26.8)	13 (18.8)	1.245	0.265
Coronary heart disease (yes), n(%)	20 (28.2)	14 (20.3)	1.181	0.277
Hypertension (yes), n(%)	25 (35.2)	17 (24.6)	1.863	0.172

**Table-II:** Comparison of surgical efficacy between two groups, n(%).

Surgical efficacy	Observation group (n=71)	Control group (n=69)	χ^2^	P
Marked effect	43(60.6)	37 (53.6)	-	-
Effective	22 (31.0)	18 (26.1)	-	-
Invalid	6 (8.5)	14 (20.3)	-	-
Total efficiency	65 (91.5)	55 (79.7)	4.006	0.045
Recurrence rate	3 (4.2)	10 (14.5)	4.379	0.036

Before surgery, there was no significant difference in MGS-GCS grading between the two groups (P>0.05). Six months after surgery, the MGS-GCS grading improvement in the observation group was significantly better than that in the control group (P<0.05) (Table-III).

**Table-III:** Comparison of consciousness states between two groups, n (%).

Time	Group	Level 0	Level 1	Level 2	Level 3	Level 4
Before surgery	Observation group (n=71)	0(0.0)	26(3.6)	22(31.0)	23(32.4)	0(0.0)
	Control group (n=69)	0(0.0)	22(31.9)	24(34.8)	21(30.4)	2(2.9)
	*Z*	0.512				
	*P*	0.609				
Six months after surgery	Observation group (n=71)	41(57.7)	14(19.7)	14(19.7)	2(2.8)	0(0.0)
	Control group (n=69)	28(40.6)	17(24.6)	19(27.5)	5(7.2)	0(0.0)
	*Z*	2.110				
	*P*	0.035				

Before surgery, mRS scores of the two groups were similar (P>0.05). Six months after surgery, the mRS scores of both groups significantly decreased compared to before intervention and were considerably lower in the observation group (P<0.05). As shown in Table-IV, there was no significant difference in the total incidence of complications between the observation group and the control group (9.9% vs. 20.3%) (Table-IV).

**Table-IV:** Changes in mRS scores and incidence of postoperative complications in two groups.

Index	Observation group (n=71)	Control group (n=69)	Z/χ^2^	P
Preoperative mRS score	3 (2-3)	3 (2-4)	-0.571	0.568
MRS score at 6 months postoperatively	1 (1-2)^#^	1 (1-2)^#^	-2.225	0.026
Complication				
Infected	3 (4.2)	5 (7.2)	0.165	0.685**
Subdural effusion	2 (2.8)	3 (4.3)	0.001	0.974**
Csf leak	1 (1.4)	2 (2.9)	0.001	0.980**
Subdural gas accumulation	1 (1.4)	4 (5.8)	0.890	0.345**
Total incidence rate	7 (9.9)	14 (20.3)	2.986	0.084*

Compared with the same group before surgery,

# P<0.05;

* Pearson’s Chi-square test;

** Fisher’s Exact Test.

## DISCUSSION

This study demonstrated that, in patients with CSDH after BCT, exhaustive drainage with urokinase flushing significantly improved surgical efficacy (91.5% vs. 79.7%) and reduced the recurrence rate (4.2% vs. 14.5%). This result is consistent with the conclusions of previous research. Ou et al.^14^ found that the recurrence rate of 1117 CSDH patients who underwent drilling craniotomy and were treated with an exhaustive drainage strategy was only 1.9% (21/1117). Zhou et al.^15^ showed that in elderly patients, urokinase-assisted exhaustive drainage after BCT was associated with a markedly reduced recurrence rate.

A study by Zheng et al.^16^ also confirmed that the recurrence rate of postoperative urokinase flushing was 2.9%, significantly lower than that of saline flushing (25.0%). Cheung et al.^17^ reported that urokinase infusion is significantly and independently associated with lower recurrence rates. The formation of CSDH is closely related to the rupture and bleeding of microvessels in the outer layer of the hematoma capsule, as well as excessive fibrinolysis.^18^ The leakage of the neovascularization in the outer membrane of the hematoma and the overexpression of the local tissue type plasminogen activator lead to the accumulation of fibrin degradation products (FDP), osmotic pressure imbalance and continuous fluid infiltration into the hematoma cavity.^19^,^20^ Since traditional 48-hour drainage cannot effectively remove the fibrous protein network and FDP, the residual substances continue to stimulate vascular permeability, becoming the pathological basis for recurrence.^19^-^21^ Urokinase, on the other hand, directly catalyzes the conversion of plasminogen into plasmin, efficiently degrades fibrin clots and capsule matrix and destroys the fibrin scaffold structure of the hematoma cavity.^18^-^21^

The results of this study showed that six months after surgery, the MGS-GCS grading and mRS score of the observation group were better than those of the control group. This result indicates that exhaustive drainage has more advantages in promoting neurological function recovery, which is consistent with the research results of Cheung et al.^17^ and Jiang N et al.^22^ It is plausible that urokinase flushing and exhaustive drainage effectively remove blood and reduce residual fluid, thereby creating a more favorable space for the recovery of brain tissue. More thorough drainage can reduce the continuous compression and stimulation of the hematoma on the surrounding brain tissue and promote the recovery of neurological function.^19^-^22^

The more pronounced improvement in MGS–GCS observed with exhaustive drainage is likely multifactorial. First, more complete evacuation reduces mass effect and intracranial pressure, which likely restores cerebral perfusion and oxygen delivery to compressed cortical regions.^17^ Second, sustained drainage with urokinase promotes brain re-expansion and limits residual subdural fluid, providing more durable decompression of cortical tissue.^18^ Third, fibrinolytic irrigation helps clear fibrin networks and hematoma fluid rich in fibrin degradation products and cytokines, thereby lowering local inflammatory stimuli that may perpetuate meningeal irritation and cortical dysfunction.^19^ Although cerebral perfusion or inflammatory mediators were not directly measured in this retrospective cohort, these mechanisms are consistent with prior experimental and clinical observations in CSDH (e.g., fibrinolysis-driven clearance, reduced recurrence, and improved functional recovery).^20^,^21^

This study did not detect a significant difference in the incidence of complications between the two surgical approaches. In drainage surgery, infections can occur due to various factors, such as the level of sterility during the surgical procedure and the management of postoperative drainage devices.^21^,^22^ Although the use of urokinase for flushing increases the number of operating steps, it is possible that if the sterile operating procedures are strictly followed during the fixed-time drainage procedure, the chance of secondary infections remains low.^23^ Moreover, the occurrence of subdural fluid accumulation, cerebrospinal fluid leakage and subdural gas accumulation is often related to surgical procedure, individual anatomical characteristics of patients and postoperative recovery,^24^ rather than the use of urokinase or continuous drainage methods. Therefore, a significant difference in the incidence of these complications between the two drainage methods is not expected. In our cohort, no cases of anaphylaxis or other severe urokinase-related adverse events were observed. All instillations were performed under aseptic conditions following the standardized protocol described in the Methods, which may have contributed to the low infection risk. These observations are consistent with prior reports on the safety of fibrinolytic irrigation in the subdural space and support the feasibility of urokinase-assisted exhaustive drainage in routine practice.

This study provides novel insights into the postoperative management of CSDH. To the best of our knowledge, few previous single-center studies have systematically compared exhaustive drainage with urokinase irrigation against fixed-time drainage following burr-hole craniostomy for CSDH, particularly when employing an individualized, imaging-based threshold for drainage cessation. Our findings indicate that exhaustive drainage can improve surgical efficacy and reduce recurrence without increasing complication rates. Clinically, these results suggest that incorporating imaging criteria and targeted urokinase irrigation into postoperative drainage protocols may help optimize patient outcomes and could be considered for wider application in similar neurosurgical settings. Strengths of this study include strict inclusion and exclusion criteria, comparable baseline characteristics between groups, and uniform surgical procedures performed by an experienced neurosurgical team, which minimized bias and enhanced internal validity. Future research should focus on multicenter, prospective trials with larger sample sizes, longer follow-up durations, and standardized neuroimaging assessment of cerebral atrophy. Incorporating data on postoperative anticoagulant and antiplatelet management will also be important to further refine and validate the proposed drainage strategy.

### Limitations:

This study supports the advantages of exhaustive drainage in the management of CSDH; however, several limitations should be acknowledged. First, this was a single-center retrospective study with a relatively small sample size, which limits the statistical power and generalizability of the findings. The six-month follow-up period may also underestimate the true recurrence and complication rates of exhaustive drainage. Future multi-center prospective studies with larger cohorts and longer follow-up are needed to validate these results. Second, exhaustive drainage may increase operative complexity and duration, requiring surgeons to have advanced technical skills and extensive experience. In elderly patients with multiple comorbidities, underlying disease progression may attenuate long-term benefits despite favorable short-term outcomes. Therefore, patient selection should be individualized: exhaustive drainage may be preferable for large, well-liquefied hematomas, whereas routine drainage may be more suitable for smaller, poorly liquefied hematomas or patients with significant comorbidities. Third, given the modest sample size and limited number of primary outcome events, we did not perform propensity score matching or multivariable regression, as these methods could introduce model instability and overfitting under insufficient events-per-variable conditions. To mitigate confounding within the current design, we applied strict inclusion/exclusion criteria, standardized perioperative protocols, and confirmed baseline comparability between groups. Future larger-scale studies should incorporate propensity score–based or multivariable adjustment strategies to further reduce bias and enhance external validity. Fourth, postoperative management of anticoagulant and antiplatelet agents could not be analyzed due to inconsistent documentation of restart timing, dosage, and duration, which were individualized according to cardiovascular or cerebrovascular comorbidities. Standardized, prospective collection of antithrombotic management data—including indication, drug class, restart timing, and dose adjustments—is warranted to clarify its impact on recurrence and complications in CSDH. Finally, cerebral atrophy was not evaluated. Preoperative neuroimaging originated from heterogeneous scanners, including those from referring hospitals, with variations in slice thickness, acquisition angles, and image quality. Many scans lacked the anatomical levels required for accurate Evans Index calculation, precluding reliable quantitative assessment. Future prospective studies using standardized imaging protocols are necessary to enable consistent evaluation of cerebral atrophy and its potential association with postoperative outcomes.

## CONCLUSION

In patients with CSDH after BCT, exhaustive drainage with urokinase flush is more efficient than the fixed-time drainage strategy, reduces recurrence and improves patient consciousness and neurological function. Further high-quality, long-term follow-up studies are necessary to more accurately evaluate the long-term effectiveness and recurrence rate of the urokinase flush drainage to provide stronger support for clinical decision-making.

### Authors’ contributions:

**JG and YX:** Study design, literature search and manuscript writing.

**HC and QG:** Data collection, data analysis and interpretation. Critical analysis.

**JG and YX:** Manuscript revision and validation and is responsible for the integrity of the study.

All authors have read and approved the final manuscript.
